# Tinkering under the Hood: Metabolic Optimisation of CAR-T Cell Therapy

**DOI:** 10.3390/antib10020017

**Published:** 2021-04-26

**Authors:** Yasmin Jenkins, Joanna Zabkiewicz, Oliver Ottmann, Nicholas Jones

**Affiliations:** 1 Institute of Life Science, Swansea University Medical School, Swansea University, Swansea SA2 8PP, UK; 952343@swansea.ac.uk; 2 Experimental Cancer Medicine Center, Department of Haematology, Heath Hospital, Cardiff University, Heath Park, Cardiff CF14 4XN, UK; zabkiewiczj1@cardiff.ac.uk (J.Z.); OttmannO@cardiff.ac.uk (O.O.)

**Keywords:** CAR-T cells, metabolism, immunometabolism, oncometabolism, tumour microenvironment

## Abstract

Chimeric antigen receptor (CAR)-T cells are one of the most exciting areas of immunotherapy to date. Clinically available CAR-T cells are used to treat advanced haematological B-cell malignancies with complete remission achieved at around 30–40%. Unfortunately, CAR-T cell success rates are even less impressive when considering a solid tumour. Reasons for this include the paucity of tumour specific targets and greater degree of co-expression on normal tissues. However, there is accumulating evidence that considerable competition for nutrients such as carbohydrates and amino acids within the tumour microenvironment (TME) coupled with immunosuppression result in mitochondrial dysfunction, exhaustion, and subsequent CAR-T cell depletion. In this review, we will examine research avenues being pursued to dissect the various mechanisms contributing to the immunosuppressive TME and outline in vitro strategies currently under investigation that focus on boosting the metabolic program of CAR-T cells as a mechanism to overcome the immunosuppressive TME. Various in vitro and in vivo techniques boost oxidative phosphorylation and mitochondrial fitness in CAR-T cells, resulting in an enhanced central memory T cell compartment and increased anti-tumoural immunity. These include intracellular metabolic enhancers and extracellular in vitro culture optimisation pre-infusion. It is likely that the next generation of CAR-T products will incorporate these elements of metabolic manipulation in CAR-T cell design and manufacture. Given the importance of immunometabolism and T cell function, it is critical that we identify ways to metabolically armour CAR-T cells to overcome the hostile TME and increase clinical efficacy.

## 1. Introduction

Chimeric antigen receptor (CAR)-T cells are currently at the forefront of cancer immunotherapy [1] and are one of the most promising treatments for advanced haematological malignancies [2,3]. CAR-T cells are genetically engineered T cells that express an artificial receptor targeting a tumour specific antigen. The generation and expansion of CAR-T cells involves either removing the patient’s autologous T cells or using healthy donor T cells (allogeneic), applying genetic modification of the T cells to express the CAR construct, and finally expanding the CAR-T cells prior to patient infusion [3,4]. During the genetic modification (via (retro)viral vector mediated delivery or other means) and expansion process, CAR-T cells are cultured ex vivo in media formulations that contain excessive, non-physiological nutrient levels [4]. Whilst the utilisation of media formulations with excessive nutrients are critical for successful CAR-T cell expansion to generate sufficient numbers for patient infusion, heightened nutrient levels may compromise the function and survival of CAR-T cells in vivo when they encounter a more hostile, nutrient-restrictive environment [5]. These metabolic challenges within the tumour microenvironment (TME) include glucose and amino acid restrictions, reduced oxygen tensions, and an abundance of immunosuppressive nutrients such as lactate and kynurenine [5,6,7]. Aside from the TME, metabolic challenges can also arise in the pre-infused CAR-T cell product, as autologous donor T cells are often collected post-treatment (chemotherapy, radiotherapy, etc.) that can inflict long-lasting metabolic defects such as mitochondrial damage [8]. Furthermore, the aforementioned treatments are accompanied with different degrees of aggressivity that is dependent on age [8]. Whilst, previous strategies for enhancing CAR-T cell therapy have focussed on enhancing T cell receptor (TCR)-related and co-stimulatory pathways, recently consideration is being given to optimising nutrient culturing conditions and strategically enhancing metabolism to better equip CAR-T cells in the TME [9].

To date, CAR-T cells have been considered a success in the treatment of haematological cancers such as B cell acute lymphoblastic leukaemia and B cell lymphomas, however, success rates are less impressive for the treatment of solid tumours [10]. Metabolic manipulation could overcome the barriers of CAR-T cell success in solid tumours and certainly warrants further investigation.

The renaissance of immunometabolism as an area of research dictates the metabolic pathways that govern immune cell fate and function [11]. Metabolic programming in T cells has come under recent scrutiny, revealing key roles in human health and disease. We now know that metabolic heterogeneity is demonstrated by the broad range of T cell compartments. For example, quiescent T cells (e.g., naïve and resting memory T cells) rely on catabolic processes such as oxidative phosphorylation (OXPHOS) to maintain housekeeping functions [12], whereas upon activation, T cells (e.g., early activated and effector T cells [13]) shift to anabolic pathways such as glycolysis (inclusive of horizontal pathways such as the pentose phosphate pathway and serine biosynthesis pathway) and the tricarboxylic cycle to generate biosynthetic intermediates required for proliferation [13,14].

This mini-review will discuss our current understanding of T cell metabolism and how this may impact the design and culture of CAR-T cells for therapeutic use. We will explore how metabolism can shape CAR-T cell memory, persistence, and effector function, and give considerations into how CAR-T cell metabolism can be modulated to optimise the efficacy of CAR-T cell therapy in the setting of solid tumours.

## 2. T Cell Metabolism and the Tumour Microenvironment

Throughout their life cycle, T cells encounter a range of energetically challenging microenvironments within the human body and are therefore challenged with the need to adapt to an altered nutrient environment dictated by cytokine/chemokine milieu and cell-cell competition in order to provide host protection [15]. All too frequently, the hostile TME [16] results in the functional impairment of immune effector cells by manipulating the metabolic programs of anti-tumour T cell populations via numerous mechanisms. One such mechanism is the direct competition for nutrients [7]. To maintain their highly energetic phenotype, cancer cells are often/typically intrinsically programmed to transport large quantities of glucose (to support aerobic glycolysis, or the ‘Warburg-effect’) and amino acids to support their exponential proliferation, thereby outcompeting local immune cell populations, including intratumoural T cells [17].

**Figure 1:**
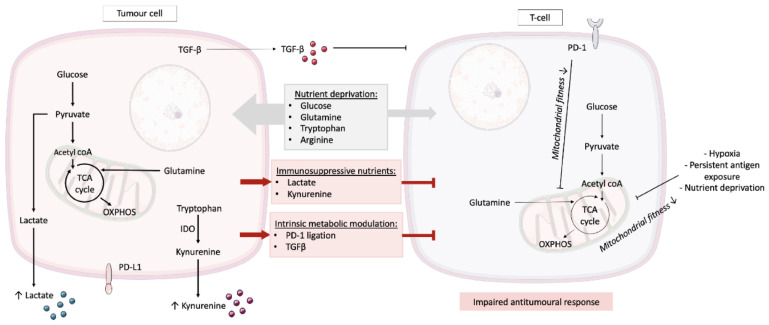
T cell metabolism and the tumour microenvironment. T cells face various metabolic challenges within the TME. Direct competition for nutrients arises such as glucose, glutamine, and amino acids, with the tumour outcompeting T cells. The tumour can also produce immunosuppressive nutrients such as lactate and kynurenine, and intrinsically modulate T cell metabolism through PD-1 checkpoint ligation and the production of TGF-β. This metabolic modulation of T cells within the TME results in an impaired anti-tumoural response. IDO, indoleamine 2,3-dioxygenase; OXPHOS, oxidative phosphorylation; PD-1, programmed cell death-1; PD-L1, programmed cell death-ligand 1; TCA cycle, tricarboxylic acid cycle; TGF-β, transforming growth factor-beta; and TME, tumour microenvironment.

Elevated levels of aerobic glycolysis by the tumour results in localised acidosis via extensive lactate production [18]. Once considered a waste product, lactate has now been shown to have both intrinsic and extrinsic effects on T cell function (Figure 1). For example, in lymph nodes, elevated lactate levels driven by low convective flow and heightened glucose metabolism inhibit T cell function to circumvent an autoimmune reaction [19]. Such parallels can be drawn between the lymph node microenvironment and TME, whereby lactate inhibits the effector T cell function [20]. Excess lactate impairs NAD+ regeneration, subsequently blocking the enzymatic reactions involving glyceraldehyde 3-phosphate dehydrogenase and 3-phosphoglycerate dehydrogenase [21]. This blockade reduces T cell proliferation, but can be rescued by restoration of the redox balance via supplementation with serine [21]. Furthermore, within the TME, lactate can promote the differentiation of naïve T cells into regulatory T cells (Tregs) whereby the carbon backbone directly enters the citric acid (TCA) cycle and contributes to energy production via OXPHOS. The ability of Tregs to thrive in a low-glucose, high-lactate TME enhances immunosuppression and tumour evasion [22] and contributes to the progression of malignancy [22]. In addition, lactate impacts on natural killer (NK) cell viability by promoting mitochondrial dysfunction leading to apoptosis [18]. Given the multiple negative implications of lactate produced by multiple cell types on anti-tumour immunity, the metabolic targeting of aerobic glycolysis is potentially an attractive therapeutic target. Indeed, the restriction of tumour glycolysis levels using the non-steroidal anti-inflammatory drug diclofenac has been demonstrated to reduce lactate levels and augment immune checkpoint therapy via the preservation of the effector T cell function [23].

Elevated aerobic glycolysis in tumour cells can have a detrimental impact on intra-tumoural T cell function (Figure 1). Glucose restriction and the limitation of the glycolytic metabolite phosphoenolpyruvate impairs/reduces calcium and the nuclear factor of activated T cells (NFAT) signalling, impairing the T cell function [17]. Studies overexpressing phosphoenolpyruvate carboxykinase 1 in T cells boosted calcium and NFAT signalling leading to restricted tumour growth in a murine melanoma model [17].

Aside from aerobic glycolysis, the expression of amino acid-metabolising enzymes by tumour cells and tumour-associated cells, deprives local T cell populations within the TME. In particular, arginine levels within the TME are reduced by tumour cells and tumour-associated macrophages (TAMs) expressing arginine metabolising enzymes such as inducible nitric oxide synthase (iNOS) and arginase, respectively. Moreover, expression by both cell types of indoleamine 2,3-dioxygenase (IDO) depletes tryptophan via the production of kynurenine, thereby further suppressing the T cell function within the TME [24] and promoting the formation of Tregs [6] (Figure 1). Glutamine is another important amino acid required for cancer cell proliferation, which is incorporated into the TCA cycle via anaplerosis where it can contribute to the generation of biosynthetic intermediates such as nucleotides, non-essential amino acids, and lipids [25]. The reliance on glutamine by multiple cancer types makes it an intriguing target therapeutically and glutamine blockade has been reported to be tolerated by effector T cells, enhancing oxidative metabolism and mitochondrial protein expression whilst proving to be detrimental for cancer cells in vivo [26].

More recently, studies have revealed that T cells within the TME experience persistent antigen exposure under hypoxic conditions leading to detrimental mitochondrial health [27,28,29]. Mechanistically, the double-edged sword of hypoxia and persistent antigen exposure impairs ADP-coupled oxidative phosphorylation, increases mitochondrial ROS production resulting in an impaired mitochondrial function leading to T cell exhaustion [28,29]. Interestingly, circumvention of this exhausted phenotype can be achieved using anti-oxidants enhancing the anti-tumour activity of chronically stimulated T cells [29].

The combination of nutrient deprivation, hypoxia, persistent antigen exposure, and immunosuppression within the TME has detrimental consequences on the mitochondrial fitness of anti-tumour T cells [16,28,30] (Figure 1). A further layer of complexity is the engagement of immune checkpoint proteins that can intrinsically modulate T cell metabolic reprogramming favouring a pro-tumour outcome [31]. For example, ligation of immune checkpoint protein programmed cell death-1 (PD-1) results in altered mitochondrial cristae morphology leading to an upregulation of fatty acid oxidation and subsequently, dysfunction in CD8+ T cells [31,32,33]. Interestingly, tumours that escape anti-PD1 blockade therapy are able to do so via the suppression of the mitochondrial function in T cells [34]. Furthermore, tumour-derived TGF-β has been shown to negatively impact on the mitochondrial function (via phosphorylation of Smad proteins), resulting in compromised IFNγ production and the reduced anti-tumour activity of T cells [16,35] (Figure 1). Collectively, the metabolic modulation of the T cell mitochondrial function appears critical to immune system evasion and cancer progression.

Therefore, for the future success of CAR-T cell therapy, particularly in a solid tumour setting it is imperative that the metabolic implications of both the tumour and the CAR-T are considered.

## 3. Current Metabolic Conditioning of CAR-T Cells

### 3.1. CAR-T Cell Design

**Figure 2:**
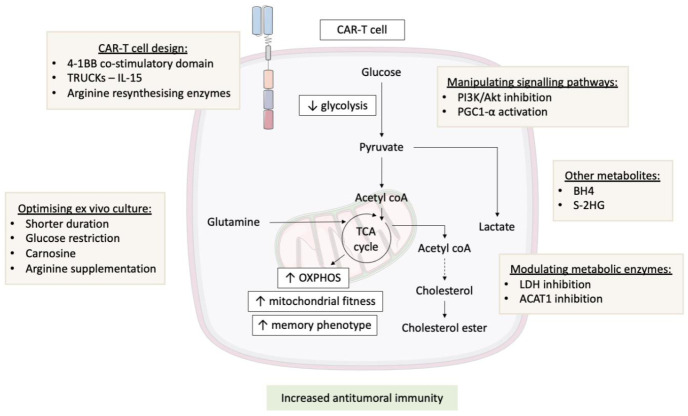
Metabolic optimisation of CAR-T cells. Various strategies are currently being investigated to enhance the metabolic program of CAR-T cells and overcome the metabolic hostility of the TME. This includes improving CAR-T cell design, optimising ex vivo culture conditions, manipulating signalling pathways, and modulating various metabolic enzymes. These strategies increase OXPHOS levels and mitochondrial fitness in CAR-T cells leading to an enhanced central memory T cell compartment, resulting in a fitter CAR-T cell product with improved anti-tumoural immunity. ACAT1, cholesterol acyltransferase 1; BH4, tetrahydrobiopterin; CAR-T cell, chimeric antigen receptor T cell; LDH, lactate dehydrogenase; OXPHOS, oxidative phosphorylation; PGC1-α, PPAR-gamma coactivator 1-α; S-2HG, S-2-hydroxygluterate; TCA cycle, tricarboxylic acid cycle; TME, tumour microenvironment; and TRUCKs, T-cells redirected for unrestricted cytokine-mediated killing.

The extracellular domain of a CAR is composed of the single-chain variable fragment of an antibody targeting an antigen expressed on the tumour cell surface, and this is combined with an intracellular T cell receptor (TCR) signalling domain. In first-generation CARs, this signalling domain is conventionally composed of the CD3ζ chain of the TCR alone, whereas successive second or third generations of CARs include one or more co-stimulatory domains, such as CD28 or 4-1BB [36] (Figure 2). CAR design itself can have an impact on CAR-T cell function, with the choice of a co-stimulatory domain governing CAR-T cell metabolism. CAR-T cells with a CD28 co-stimulatory domain (Yescarta) show enhanced aerobic glycolysis and effector memory differentiation [37]. Whereas the inclusion of a 4-1BB co-stimulatory domain rather than CD28 (Kymriah, UCART19, Liso-cel) promotes an oxidative metabolism and increases mitochondrial biogenesis [37]. This metabolic reprogramming results in enhanced central memory differentiation and improves CAR-T cell proliferation and persistence in vitro. The importance of co-stimulatory domain selection is evidenced by the differential downstream metabolic programs they govern. For instance, one particular co-stimulatory domain may be more beneficial at treating a certain malignancy with a particular metabolic program. Furthermore, consideration should be given towards the quality of downstream signal transduction (low or high affinity binding) and its influence on downstream CAR-T cell metabolism. Identifying differences in signal transduction and subsequent metabolic reprogramming may give insight into CAR-T cell functional differences and exhaustion profiles with therapeutic value.

Next generation (fourth generation) CAR-T cells are known as T-cell redirected for unrestricted cytokine-mediated killing (TRUCKs) and incorporate a transgenic cytokine release system designed to improve proliferation and persistence in vivo [38] (Figure 2). Typically, the cytokines incorporated include IL-12, IL-15, and IL-18, most of which also have known metabolic effects on T cells. For example, CAR-T cells expanded in the presence of IL-15 have decreased mTORC1 activity, which results in a reduction in glycolysis. IL-15-treated CAR-T cells also displayed elevated levels of OXPHOS and enhanced spare respiratory capacity, as well as an increased expression of fatty acid oxidation-related genes. These metabolic changes result in less differentiated CAR-T cells that maintain a stem cell memory phenotype and enhance CAR-T cell proliferation, improving their in vivo anticancer activity and longevity [39].

Whilst a specific CAR-T cell design has clear implications on the metabolic program of the product, another important consideration is how these CAR-T cells are subsequently expanded and maintained prior to patient infusion.

### 3.2. Optimising Media

One of the major factors limiting the efficacy of CAR-T cell therapy is the metabolic hostility of the TME. CAR-T cells face the same metabolic challenges and immunosuppression within the TME as host T cells, having to compete with tumour cells for nutrients and overcome suppression by various metabolites. In addition, the suppressive TME may initially impact on the homing capacity of CAR-T cells. Enhancing CAR-T cell memory is key to improving their persistence and anti-tumour activity and increasing their therapeutic capacity. CAR-T cells are currently created, expanded, and maintained in media formulation containing excessive nutrient levels such as carbohydrates and amino acids [4,9]. In particular, two supraphysiological nutrients are glucose and glutamine, which are essential fuels for rapidly proliferating, activated immune cells [13,40]. Clinical and experimental media formulations do not (i) recapitulate the nutrient hostile, immunosuppressive environment of the TME, including any abundant oncometabolites or (ii) rescue any T cells with long-lasting metabolic defects inflicted from pre-cancer treatments such as chemotherapy and radiotherapy.

Efforts have been made to move away from the reliance on human serum in media used for CAR-T cell manufacturing with the identification of a concentrated growth factor derived from human transfusion grade whole blood fractions, known as PhysiologixTM xeno-free (XF) hGFC (Phx) [9]. Phx, in comparison to human serum, has been demonstrated to enrich carnosine levels that enhance downstream T cell proliferation and peripheral blood abundance in both clinical and research grade media. Furthermore, the authors demonstrate that carnosine inclusion in media formulations are able to neutralise extracellular protons (H^+^) derived from lactate (via aerobic glycolysis), promoting a metabolic transition from glycolysis towards an oxidative phenotype in CAR-T cells, thus supporting its inclusion in media formulation [9]. Interestingly, previous methods that curtail T cell glycolysis levels ex vivo promote anti-tumour activity, highlighting the potential importance of including carnosine in CAR-T cell clinical culture methods [41]. A summary of optimising ex vivo culture can be found in Figure 2.

Moreover, shortening the duration of CAR-T cell expansion ex vivo has also been shown to limit their differentiation resulting in CAR-T cells with an enhanced proliferation and effector function in vitro. Importantly, these CAR-T cells also displayed enhanced persistence and improved antileukemic activity in a mouse acute lymphoblastic leukaemia (ALL) xenograft model [42]. It is clear that in vitro expansion and culture of CAR-T cells considerably impacts on their phenotype and function. To counteract this, efforts are being made to generate in vivo CAR-T cells, thus removing the non-physiological culture conditions in vitro CAR-T cells are exposed to [43]. For instance, lentiviral vectors can be delivered specifically to CD8+ T cells in vivo, generating CD19-CAR-T cells with effective B-cell elimination [44].

### 3.3. Lactate

Lactate is one of the most abundant metabolites within the solid TME with levels reaching up to a staggering 40 mM [45]. As discussed previously, lactate has detrimental effects on the immune system, that ultimately reduces the anti-tumour response, specifically promoting the differentiation of Tregs [22]. Whether contributions of lactate within the TME can promote the formation of a CAR-Treg phenotype, compromising the anti-tumour response is yet to be determined.

The recently-developed method of in vivo metabolomics by Ma et al., could potentially identify the contribution of lactate-derived carbons towards central metabolic pathways in CAR-T cells [46]. Given the reported differences of T cell metabolism between in vitro and in vivo, the technique of in vivo metabolomics could be used to inform downstream methods of optimising CAR-T cell metabolism for therapeutic success [46].

Whilst little is known about the direct influence of lactate on CAR-T cell metabolism and function, in an attempt to overcome the suppressive properties of lactate, studies have begun to investigate the mechanistic effect of lactate dehydrogenase (LDH) inhibition. Specifically, the inhibition of LDH enhances the CAR-T cell function [47,48], in particular one study identified a synergistic effect with IL-21 to promote CAR-T memory and enhanced downstream anti-tumour immunity [48].

### 3.4. Arginine

CAR-T cell metabolism may also be manipulated to overcome low arginine levels within the TME.

Supplementing the media with L-arginine during ex vivo culture also has the potential to improve CAR-T cell therapy [49] via increased intracellular levels, driving a shift in CAR-T cell metabolism from glycolysis towards OXPHOS. This metabolic shift promotes the formation of central memory-like T cells, with enhanced survival and improved anti-tumour activity in vivo. Another method developed to overcome limited in vivo arginine levels is the engineering of CAR-T cells to express the arginine resynthesizing enzymes argininosuccinate synthase (ASS) and ornithine transcarbamylase (OTC). These modifications alongside the CAR itself can enhance the proliferation and improved anti-tumour activity in mouse models [24].

### 3.5. Glucose Restriction

To date, most studies have reported an association between elevated OXPHOS and the enhanced formation of the central memory CAR-T cell compartment leading to improved treatment outcomes [50,51]. Therefore, methods to boost CAR-T cell OXPHOS levels both ex vivo and in vivo have been under scrutiny [52,53]. Interestingly, glucose restriction via the substitution of glucose for another carbohydrate such as galactose in cell culture media has previously been demonstrated to boost OXPHOS levels in T cells and NK cells [40,54]. Furthermore, a recent study has investigated a period of glucose restriction after activation during CAR-T cell expansion as a potential strategy to enhance the efficacy of CAR-T cell therapy [55]. Antigen-specific CD8+ effector T cells subjected to transient glucose restriction (TGR) displayed enhanced tumour clearance in a mouse model of lymphoma, with an increase in the number of donor CD8+ T cell in circulation that persisted up to 20 days after adoptive transfer. Additionally, CAR-T cells subjected to TGR displayed an increased production of the effector molecules IFNγ and granzyme B [55]. This enhanced persistence and anti-tumour activity is underpinned by metabolic rewiring, with TGR CD8+ effector T cells undergoing a metabolic shift towards OXPHOS and showing an enhanced anabolic phenotype upon re-exposure to glucose.

A further consequence of glucose restricted T cells is the ability to utilise inosine [56]. T cells exhibit metabolic plasticity during glucose restriction, incorporating inosine-metabolised carbons into central metabolic pathways to generate adenosine triphosphate and biosynthetic intermediates resulting in an enhanced oxidative metabolism [56].

Whether TGR is clinically relevant is yet to be determined, however there are multiple considerations as to how this could be achieved. Given that glucose is essential to generate clinically sufficient numbers of CAR-T cells for infusion, it is unlikely that an early period of TGR would be a success. However, whether the introduction of TGR (for example with a galactose substitution) prior to infusion would generate a therapeutic avenue is an interesting thought. Furthermore, whether this method could be further refined by utilising a mixed culture system, with half the CAR-T cells cultured under standard clinical conditions and half under TGR to generate a fitter product remains to be seen.

### 3.6. Other Metabolites

It is likely that multiple metabolites could influence the generation of a fitter CAR-T cell product in the short- and long-term. Glutamine is a critical, non-essential amino acid required for T cell function [13], and whilst it is thought that amino acid competition exists between the tumour and T cells [5], little is known about the role of glutamine in CAR-T cell culture and function. A study recently described a novel glutamine antagonist, JHU083, that suppresses both glycolytic and oxidative metabolic pathways in tumour cells, leading to a reduction in hypoxia, acidosis, and nutrient depletion [26]. Surprisingly, glutamine blockade using JHU0833 promoted elevated effector T cell oxidative metabolism and longevity within the TME [26]. This important study highlights that intratumoural T cells are able to demonstrate metabolic plasticity with regards to glutamine metabolism becoming dispensable and would therefore certainly be an interesting avenue to pursue in combination with CAR-T cells [26]. Furthermore, T cells cultured ex vivo in glutamine-depleted media demonstrated robust anti-tumour activity upon adoptive transfer in mice. These studies highlight the requirement for investigation into the role of glutamine manipulation in CAR-T cell culture conditions pre-infusion [57].

The S-enantiomer of 2-hydroxygluterate (S-2HG) is an immunometabolite that is produced following HIF-1α stabilisation. S-2HG has a role in regulating T cell fate, inhibiting their differentiation into effector cells. Expanding CAR-T cells in the presence of S-2HG yields increased proportions of central memory cells, regardless of the co-stimulatory domain included in the CAR design. Importantly, the in vitro cytotoxic activity and cytokine production of S-2HG treated CAR-T cells was not impaired, and in a mouse xenograft model, CAR-T cells treated with S-2HG display enhanced anti-tumour activity and reduced tumour progression [58].

Another beneficial metabolite identified, tetrahydrobiopterin (BH4), can be produced by activated T cells and has a role in the modulation of iron metabolism and mitochondrial bioenergetics [59]. Intriguingly, kynurenine-mediated T cell immunosuppression can be rescued by BH4, outlining its importance in anti-tumour immunity and raising further questions about its inclusion in CAR-T cell design [59].

Whilst the primary focus of most studies revolves around the interplay of glucose and amino acid metabolism between the tumour and the CAR-T cells, another important biomolecule to consider is cholesterol. Cholesterol is an integral component of the cell membrane, essential for T cell proliferation [60]. The inhibition of cholesterol esterification by the enzyme cholesterol acyltransferase 1 (ACAT1) utilising the repurposed drug avasimibe leads to an improved anti-tumour response by CD8+ T cells that further enhanced outcomes in combination therapy with anti-PD-1 treatment [61]. Such findings have now been translated into the CAR-T cell field, whereby siRNA targeting of ACAT1 in a mesothelin-expressing pancreatic carcinoma model reduced tumour volume, elevated IFNγ levels, and improved overall cancer targeting in vivo [62]. Repurposing avasimibe (atherosclerosis treatment, clinically) as a combination therapy with CAR-T cells is an attractive avenue given the known safety profiles in humans [61].

### 3.7. Manipulating Signalling Pathways

Successive generations of CAR-T cells have been designed to incorporate downstream signalling enhancement. One pathway of interest, the PI3K/Akt signalling pathway, has previously been implicated in upregulating glycolysis levels in stimulated human T cell subsets [13,14]. In a CD33 targeted CAR-T cell model of acute myeloid leukaemia, the authors identified upregulation of PI3K/Akt/mTOR and glycolytic gene expression in ex vivo expanded T cells in comparison to control T cells.

The PI3K/Akt/mTOR pathway is associated with a terminal effector T cell differentiation that can hinder CAR-T cell success in comparison to the central memory phenotype [63] (Figure 2). As decreasing T cell glycolysis levels ex vivo is associated with a fitter T cell product in vivo, in an attempt to circumvent this, the authors utilised a panel of inhibitors targeting PI3K, Akt, mTOR, and the glycolysis pathway [50]. Here, the authors observed increased naïve and central memory T cell numbers with largely decreased terminal effector cells. Whilst the inhibitors employed had a positive effect on the desired T cell compartments, only PI3K inhibition maintained cell numbers in comparison to the control. In corroboration with the PI3K inhibitor, another study investigated the role of Akt inhibition in depth. Here, the authors elegantly show that Akt inhibition elevates FOXO1 expression resulting in heightened CD62L-expressing central memory T cells. These findings were accompanied with a reduced glycolytic gene signature and lowered glycolysis levels in a human’s anti-CD19 CAR-T cells [64].

Small-molecule inhibitors of PI3K have failed to enter the clinic for a range of cancers due to limited stability, poor specificity, and high toxicity, and are therefore unlikely to compliment CAR-T cell therapy in vivo, however it could be an ex vivo avenue for CAR-T cell optimization [65,66].

Akt along with other signalling molecules such as AMP-activated protein kinase (AMPK), p38 MAPK, and SIRT1 can post-transcriptionally modify the PPAR-gamma coactivator 1-α (PGC1-α) [16]. These modifications can increase mitochondrial biogenesis and OXPHOS levels. Furthermore, T cells with an enforced expression of PGC1-α, improve mitochondrial biogenesis, cytokine secretion, and intratumoural T cell function in vivo, counteracting the impaired mitochondrial OXPHOS observed in dysfunctional intratumoural T cells [16]. Potential activation of PGC1-α is already implemented in CAR-T cell design, as an engagement of the co-stimulatory molecule, 4-1BB has been demonstrated to enhance PGC1-α levels dependent on p38-MAPK in T cells [67]. Methods that can further enhance levels of PGC1-α activation certainly warrant investigation in CAR-T cell design (Figure 2).

## 4. Concluding Remarks

It is known that the TME can shape the metabolic and phenotypic fitness of CAR-T cells. This hostile, immunosuppressive microenvironment leads to an unfavourable response when utilising CAR-T cells. Therefore, investigating the metabolic and phenotypic fitness of CAR-T cells in response to the TME is essential in order to improve the efficacy of CAR-T cells. It is now clear that there are many ways that CAR-T cell metabolism can be manipulated for therapeutic gain. Multiple in vitro studies have reported that boosting oxidative metabolism and mitochondrial fitness leads to an increased central memory T cell compartment resulting in a fitter CAR-T cell product and improved outcomes, however many of these strategies are yet to be tested in clinical trials. Furthermore, questions still remain about the impact of the CAR-T cell manufacturing process ultimately on the end product’s metabolism, for example expansion in the presence of various cytokines such as IL-2, IL-7, or IL-15, the use of bioreactors vs. bags, the type of stimulus utilised to activate the modified T cells, implications of using peripheral blood mononuclear cells or individually isolated T cell populations, and whether using antigen specific T cells stifles any metabolic heterogeneity.

Current CAR-T cell design has incorporated the three traditional signals of T cell activation (signal 1; TCR, signal 2; co-stimulatory, and signal 3; cytokine stimulation) with somewhat unintentional effects on CAR-T cell metabolism. However, whether future generations of clinical CAR-T cell design could include a metabolically orientated ‘fourth signal’, such as overexpression of particular nutrient transporters or metabolic enzymes with the hope of generating a fitter product, is of particular interest. An additional factor to consider would be CAR-T cell therapy in combination with a metabolic modulator to potentially further improve efficacy. Whilst there are a multitude of metabolic modulators to pursue, considerable attention is required to demonstrate no detrimental effects on the CAR-T cells compromising function. We envisage the identification of therapeutic windows that could be developed to circumvent any interfering effects.

The future of next generation CAR-T cell therapies is an exciting area for the treatment of malignancy. Given such promising results in haematological cancers such as B cell lymphomas [68], there is much to look ahead to with regards to the treatment of solid tumours. The re-emergence of the fields of immunometabolism and oncometabolism over the last 10–15 years provide us with a platform to further enhance CAR-T cell therapy and improve clinical outcomes in the fight against cancer.

## Data Availability

Not applicable.
